# Targeting IL-22 and IL-22R protects against experimental osteoarthritis

**DOI:** 10.1038/s41423-020-0491-y

**Published:** 2020-07-07

**Authors:** Changhua Yi, Yongxiang Yi, Jie Wei, Qingwen Jin, Junwei Li, Pradeep Kumar Sacitharan

**Affiliations:** 1 Department of Infectious Diseases, The Second Hospital of Nanjing, The Affiliated Hospital of Nanjing University of Chinese Medicine, #1 Zhongfu Road, Nanjing, Jiangsu Province China; 2 College of Medical Laboratory, Shaoyang University, ShaoYang, 422000 Hunan Province China; 3 Department of General Surgery, The Second Hospital of Nanjing, The Affiliated Hospital of Nanjing University of Chinese Medicine, #1 Zhongfu Road, Nanjing, Jiangsu Province China; 4 Department of Neurology, The Sir Run Run Hospital, Nanjing Medical University, #109 Longmian Avenue, Jiangning District, Nanjing, Jiangsu Province China; 5 College of Veterinary Medicine, Qingdao Agricultural University, 266109 Qingdao, China; 6 The Institute of Ageing and Chronic Disease, University of Liverpool, Liverpool, L7 8TX UK; 7 Department of Biological Sciences, Xi’an Jiaotong-Liverpool University, #111 Ren’ai Road, Suzhou Industrial Park, Suzhou, 215123 Jiangsu Province China

**Keywords:** Interleukins, Target identification

Osteoarthritis (OA) is characterized by cartilage degradation, pain, and synovitis.^1^ Joint inflammation driven by cytokines has been demonstrated to cause cartilage degradation and pain.^2^ However, approaches to neutralize cytokines, such as IL-1 and TNF-α, known to be involved in OA have shown poor clinical efficacy.^3^ There is an unmet clinical need to find better anti-inflammatory and pain targets for OA therapy and to elucidate the role of other cytokines in OA pathogenesis. Previous studies have shown that IL-22 and its receptor IL-22R play central roles in inflammation and diseases such as psoriasis, ulcerative colitis, graft-versus-host disease, certain infections and tumors, as well as in liver and pancreas damage.^4,5^ The role of IL-22/IL-22R and the potential for therapeutic targeting of both proteins in OA remain largely unknown, which we sought to investigate.

We first examined human OA tissues to investigate whether IL-22/IL-22R expression levels change in disease. IL-22 was increased in the synovial fluid (SF) but not in the sera of OA patients compared to non-OA patients (Fig. 1a, b). Protein and mRNA expression of IL-22R was elevated in human chondrocytes isolated from OA patients (Fig. 1c–e). However, IL-22 protein and mRNA expression was only increased in fibroblast-like synoviocytes (FLS) isolated from OA patients (Fig. 1f–h). The increased concentration of IL-22 in the SF but not in the sera of OA patients seems to suggest that this cytokine plays a local role in OA joints with tissue-specific expression. It seems plausible that IL-22 produced by FLS in OA joints is important for disease progression. FLS-produced IL-22 may act mainly on IL-22R on chondrocytes, as indicated by the elevated expression levels of the receptor in human OA chondrocytes. Although IL-22/IL-22R have been reported to be increased in inflamed OA synovium and linked with increased protease expression,^6,7^ further studies investigating the precise downstream signaling of IL-22/IL-22R in chondrocytes need to be conducted.

**Fig. 1:**
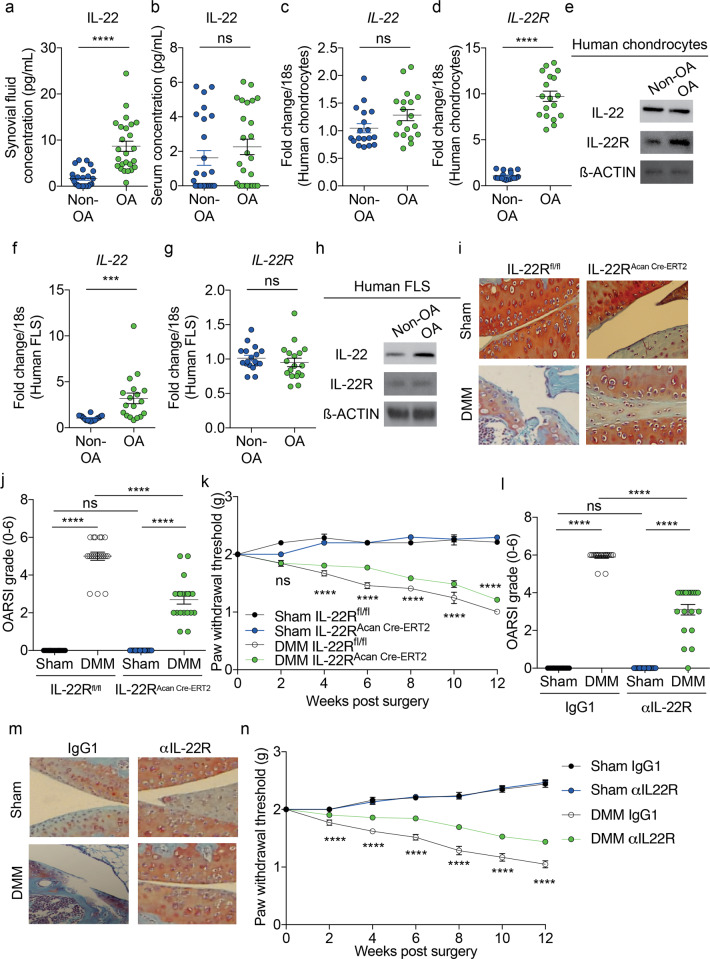
Targeting IL-22 signaling protects against experimental osteoarthritis. IL-22 concentration (pg/mL) in **a** SF and **b** serum of non-OA and OA patients (*n* = 25). **c** IL-22 mRNA, **d** IL-22R mRNA and **e** IL-22 and IL-22R protein expression in isolated human chondrocytes from non-OA and OA patients (*n* = 18). **f** IL-22 mRNA, **g** IL-22R mRNA and **h** IL-22 and IL-22R protein expression in isolated FLS from non-OA and OA patients (*n* = 18). **i**, **j** OARSI scoring of cartilage and **k** von Frey pain assessment of sham- or DMM-operated IL-22R^fl/fl^ control mice and IL-22R^Acan Cre-ERT2^ mice (12 weeks post surgery end timepoint) (*n* = 20). **l**, **m** OARSI scoring of cartilage and **n** von Frey pain assessment from sham- or DMM-operated WT mice treated i.a. with either IgG1 (control; 50 µg per mouse; 3 times per week for 12 weeks post surgery) or αIL-22R (50 µg per mouse; 3 times per week for 12 weeks post surgery) (*n* = 20). All RT-qPCR gene expression levels were normalized to the endogenous level of 18S in the respective groups. Data are expressed as the mean ± SEM with two-tailed *t*-test or two-way analysis of variance followed by the Tukey-Kramer test or repeated measures 2-way ANOVA with Bonferroni’s post hoc tests. *n* indicates the number of human specimens or mice per group. NS nonsignificant. ***p < 0.001 or ****p < 0.0001 is represented in all figures

Having observed the tissue-specific increase in IL-22 in chondrocytes and IL-22R in FLS from OA patients, we next investigated whether these results had an in vivo relevance during disease pathogenesis. We tested this hypothesis first by successfully generating inducible IL-22R chondrocyte-specific KO mice (IL-22R^Acan Cre-ERT2^) (Supplementary Fig. S1a, b). IL-22R^Acan Cre-ERT2^ mice displayed decreased cartilage degradation, synovitis, osteophyte maturity and pain compared to IL-22R^fl/fl^ control mice post experimental OA (surgical destabilization of the medial meniscus (DMM)) (Fig. 1i–k and Supplementary Fig. S1c–e). We also successfully generated inducible IL-22 FLS-specific KO mice (IL-22^Col1a2 Cre-ERT2^) (Supplementary Fig. S2a, b). IL-22^Col1a2 Cre-ERT2^ mice demonstrated reduced disease outcomes and pain compared to IL-22^fl/fl^ control mice post DMM surgery (Supplementary Fig. S2c–h). To our knowledge, this is the first set of in vivo data that show, using tissue-specific KO mice, the pathogenic role of IL-22/IL-22R in both OA disease progression and OA-related pain. IL-22 in RA joints has both beneficial^8^ and pathogenic^9^ roles; together with our results, this may suggest that IL-22 is part of divergent inflammatory responses orchestrated by different joint cells in OA compared to RA.

Next, we wanted to investigate whether therapeutically neutralizing IL-22 and IL-22R may attenuate OA in vivo. WT mice treated with an IL-22R neutralizing antibody demonstrated decreased cartilage degradation, synovitis, osteophyte maturity and pain compared to IgG1-treated control mice post DMM surgery (Fig. 1l, m and Supplementary Fig. S3a–c). Similarly, WT mice treated with an anti-IL-22 antibody displayed reduced disease outcomes (Supplementary Fig. S4a–f). Our in vivo studies also showed the possible potential of using anti-IL-22/IL-22R antibodies to treat OA and its related pain. A number of basic studies and clinical trials have shown the benefits of targeting IL-22/IL-22R in systemic immune diseases.^4^ Our study indicates a further benefit of administering anti-IL-22/IL-22R in the joint to avoid any adverse systemic effects.^10^

Together, our data reveal the cell-specific pathogenic role of IL-22 (FLS specific) and IL-22R (chondrocyte specific) in OA. Targeting both IL-22 and IL-22R seems to be a plausible treatment option for OA and pain.

## Supplementary information

Supplemental Material
