# Supplementary material for: Targeting IL-22 and IL-22R protects against experimental osteoarthritis
Source: Cell Mol Immunol. 2020 Jul 7;18(5):1329–31. doi: 10.1038/s41423-020-0491-y (PMC8093291; doi:10.1038/s41423-020-0491-y)
Supplement: Supplementary file 1 — Supplemental Material [file 41423_2020_491_MOESM1_ESM.docx]

**Supplementary Material**

**Materials and Methods**

**Human samples**

Non-OA or OA knee/hip cartilage, synovial fluid (SF) and serum were obtained from patients at The Second Hospital of Nanjing. Non-OA samples were obtained from patients who had undergone surgery for tumours of the lower leg. Tissue samples were collected with informed donor consent (written) in full compliance with Declaration of Helsinki and approval of the Ethics Committee of The Second Hospital of Nanjing. All samples were transferred to the laboratory within 2 hours. Serum was obtained from whole blood by centrifugation at 1600g for 15 minutes at 20°C. SF was centrifuged for 20 minutes at 3,000g. Serum and SF were stored at −80°C before experimentation.

**Isolation and culture of human chondrocytes**

Cartilage was excised into pieces and incubated overnight in Dulbecco's modified Eagle's medium (DMEM; Lonza) with 1mg/mL collagenase A (Roche Pharmaceuticals) at 37°C for 5-6 h to isolate chondrocytes. Isolated human chondrocytes were cultured in DMEM media containing 4.5g/l of glucose and L-glutamine (Lonza). DMEM was supplemented, unless stated otherwise with 10% fetal calf serum (FCS; Lonza), 1% Penicillin and Streptomycin (Lonza), Amphotericin B (Gibco) and 2% (4-(2-hydroxyethyl)-1-piperazineethanesulfonic acid solution (Lonza). Cells were maintained under sterile conditions in a humidified atmosphere of 37°C containing 95% oxygen and 5% (v/v) carbon dioxide unless stated otherwise. Cells used in experiments were at passage 1.

**Isolation and culture of human fibroblast like synoviocytes (FLS)**

Synovium tissue samples were minced into pieces and treated for 4 hours with 4 mg/ml of collagenase A in DMEM at 37°C in 5% CO2. Dissociated cells were then centrifuged at 500g, resuspended in DMEM supplemented with 10% FCS, 2 mM L-glutamine, 1% Penicillin and Streptomycin and plated. After overnight culture, the nonadherent cells were removed, and the adherent cells were cultured in DMEM supplemented with 20% FCS. The cultures were kept at 37°C in 5% (v/v) carbon dioxide, and the medium was replaced every 3 days. When the cells approached confluence, they were passaged after dilution (1:3) with fresh medium. FLS from passages 4–5 were used in each experiment. The cells were morphologically homogeneous and exhibited the appearance of synovial fibroblasts.

**RNA analysis**

Total RNA was isolated from human cell cultures using RNeasy mini kit (Qiagen) and reverse transcribed using a High Capacity reverse transcription cDNA kit (Applied Biosystems) according to the recommendations of the manufacturer. RT-qPCR was carried out on a Real-Time PCR System (Zeesan systems). Relative gene expression was analysed by the ∆∆Ct method using 18S as an endogenous control gene and TaqMan probes (Table 1):

**Table 1:** TaqMan probes used in RTq-PCR experiments

| **Gene** | **Human** | **Mouse** |
| --- | --- | --- |
| *18S* | Hs03003631_g1 | Mm02601776_g1 |
| *IL-22* | Hs01574154_m1 | Mm01226722_g1 |
| *IL-22R* | Hs00364814_m1 | Mm01192943_m1 |

**Western blot analysis**

Tissue or cell samples were homogenized in lysis buffer (RIPA buffer) (Sigma-Aldrich), Ethylenediaminetetraacetic acid (EDTA)-free protease inhibitor (Roche Pharmaceuticals), Phosphatase inhibitor cocktail 2 and 3 (Sigma-Aldrich) and protein levels quantified by bicinchoninic acid (BCA) assay (Thermo Fisher Scientific). Samples were probed overnight with primary antibody: IL-22 (~17 kDa; 1:1000; Novus Biologicals; NBP2-41245), IL-22R (~64 kDa; 1:1000; Novus Biologicals; NBP1-76724) and β-actin (loading control) (~42 kDa; 1:20,000; Sigma-Aldrich; A2228).

**Mice**

All animal procedures were approved by local ethics committee. Mice weighed 18 – 25g and were maintained in cages under controlled temperatures (19 – 23°C) and lights (12 h light, 12 h dark cycle). All mice had *ad libitum* access to water and chow.

**Generation of FLS and cartilage specific IL-22 or IL-22R conditional KO mice**

Mice carrying a floxed IL-22 allele (IL-22^fl/fl^; Taconic) or IL-22R allele (IL-22R^fl/fl^; Jackson Laboratory) were bred with homozygote mice carrying transgenes for COL1A2 Cre-ERT2 (Jackson Laboratory) or Aggrecan Cre-ERT2 (Jackson Laboratory). Hence, we generated two different types of homozygote double transgenic tissue specific conditional knockout (KO) mice. (1) IL-22^fl/fl^; COL1A2 Cre-ERT2 (IL-22^COL1A2 Cre-ERT2^) which are FLS specific IL-22 conditional KO mice. (2) Aggrecan Cre-ERT2 (IL-22R^Acan Cre-ERT2^) which are cartilage specific IL-22R conditional KO mice. Mice were obtained at the expected Mendelian ratio with no adverse phenotypic side effects. Adult mice (8 weeks of age) were administered intraperitoneal doses of free base tamoxifen (TX) (2 mg/kg; Sigma-Aldrich) three times every other day for one week to induce conditional KO of IL-22 or IL-22R. IL-22^fl/fl^ or IL-22R ^fl/fl^ mice were used as controls for conditional KO mice.

**Experimental OA**

Murine OA was induced by using the destabilization of the medial mencius (DMM) model. 10-week-old male mice were anaesthetized. The medial menicus was identified and the attachments of its anterior horn to the tibial plateau was cut. For sham control limbs, the mice were anaesthetized and prepared as before. The right knee was opened using the same medial para-patellar approach and the meniscus identified but the menisco-tibial ligament was not released. The incision was closed, and the mice were left to recover.

**Pain assessment**

Pain in mice was measured using the von Frey test and the hot-plate assay. Pain tests were conducted three times before sham or DMM surgery and then once every two weeks after surgery. For the hot plate assay a surface of 55 ± 0.5°C was used with a latency period (cut-off) of 30 s defined as complete analgesia. For the von Frey test mice were allowed to acclimate for at least 15 min before mechanical allodynia was tested by touching the plantar surface of the hind paw with von Frey filaments in ascending order of force for up to 6 s.

***In vivo* treatments**

Mouse αIL-22 (50 µg per mouse; PeproTech) or mouse αIL-22R (50 µg per mouse; R&D Systems) antibodies were dissolved in sterile PBS and injected intra articularly (i.a.) in respective groups of mice 3 times a week. Isotype control IgG1 (Sigma-Aldrich) was dissolved in sterile PBS and injected via i.a. in mice at the dose of 50 µg per mouse.

**Histology**

Knee joints were harvested by excising at the proximal femur and distal tibia. The skin and surrounding muscles were then removed without disturbing the joint and its associated ligaments. Knee joints were fixed in 10% neutral buffered formalin for at least 24 h before any further processing. The specimens were decalcified in 20% formic acid for 14 days. The knees were then processed as per standard protocol: 2 changes of formalin (40°C) followed by 70% alcohol (40°C), 90% alcohol (40°C), 3 changes of absolute alcohol (40°C), 4 changes of xylene (40°C) and 4 changes of paraffin wax (60°C) with each change lasting 1.5hrs. The knees were then embedded in paraffin wax. 4μm thick coronal sections were cut and stained with Safranin-O.

**Disease scoring**

Cartilage destruction was scored by blinded observers using the OARSI grading system [6]. Synovitis was determined by grading synovial inflammation (grade 0–3) as previously described [7]. Osteophyte maturity (grade 0–3) was measured as previously described [8].

**Extraction of murine cartilage protein to validate knockdown of IL-22R protein expression in conditional cartilage specific KO mice**

Mice were injected with TX at 5 weeks of age and culled at week 7. Young mice were used because hips at 7 weeks old is still cartilaginous in nature. Hips were avulsed and placed in ice cold protein lysis buffer (Sigma-Aldrich; see western blot method). Thereafter, samples were shaken for 2 h at 4°C. The samples were then centrifuged at 13,000 × g for 5 min at 4°C and the supernatants were collected for western blotting experiments.

**Extraction and culture of murine FLS to validate knockdown of IL-22 in conditional FLS specific KO mice**

Mice were injected with TX at 8 weeks of age and culled at week 10. The articular cavity murine knee joints were cut open along both sides of the patella under a microscope to isolate the intra‑articular synovium carefully. Thereafter, the connective tissues around the synovium were carefully eliminated under a microscope. The synovium was finely chopped in DMEM media and 0.5 ml 1% type IV collagenase (Thermo Fisher Scientific) and incubated at a constant temperature of 37˚C in an orbital shaker incubator (200 rpm) for 60 min. Samples were vortexed vigorously for 1.5 min to release the cells. The samples were centrifuged for 5 min at 3000 x g and resuspended with DMEM supplemented with 10% FCS and 1% penicillin‑streptomycin. The cells were seeded and cultured (37˚C, 5% CO2) until confluence for RNA and protein extraction. Intra‑articular synovium of 10 murine knee joints had to be pooled together for 1*n*.

**RNA analysis of murine tissues to validate conditional KO mice**

Mice were injected with TX at 8 weeks of age and culled at week 10. Validation of efficient knockdown of IL-22 and IL-22R mRNA expression in conditional KO mice was performed on microdissected cartilage pooled from 4 mouse knee joints (1*n*) or isolated FLS cells. Microdissected cartilage was finely homogenised and total RNA was isolated using a RNeasy micro kit (Qiagen). Whereas, total RNA was isolated from cultured murine FLS cells using the RNeasy mini kit (Qiagen).

**ELISA**

Stored supernatants or SF were defrosted at room temperature and IL-22 concentrations were measured via ELISA (Abcam; ab119543). Assays were conducted as per the manufacturer’s instructions.

**Statistical analysis**

All data are expressed as mean ± standard error of mean (S.E.M) of n observations. Experiments were statistically analysed utilising the Students unpaired t-test or analysed with two-way analysis of variance followed by the Tukey-Kramer test or repeated measures 2-way ANOVA with Bonferroni’s post hoc tests. A significant difference was accepted when p< 0.05, p< 0.01, p<0.001 or p< 0.0001 represented in all tables and figures as *, **, *** or **** respectively. Data analysis was performed using GraphPad Prism® 5.0 (GraphPad Software, California, U.S.A).

**Supplementary Figures**


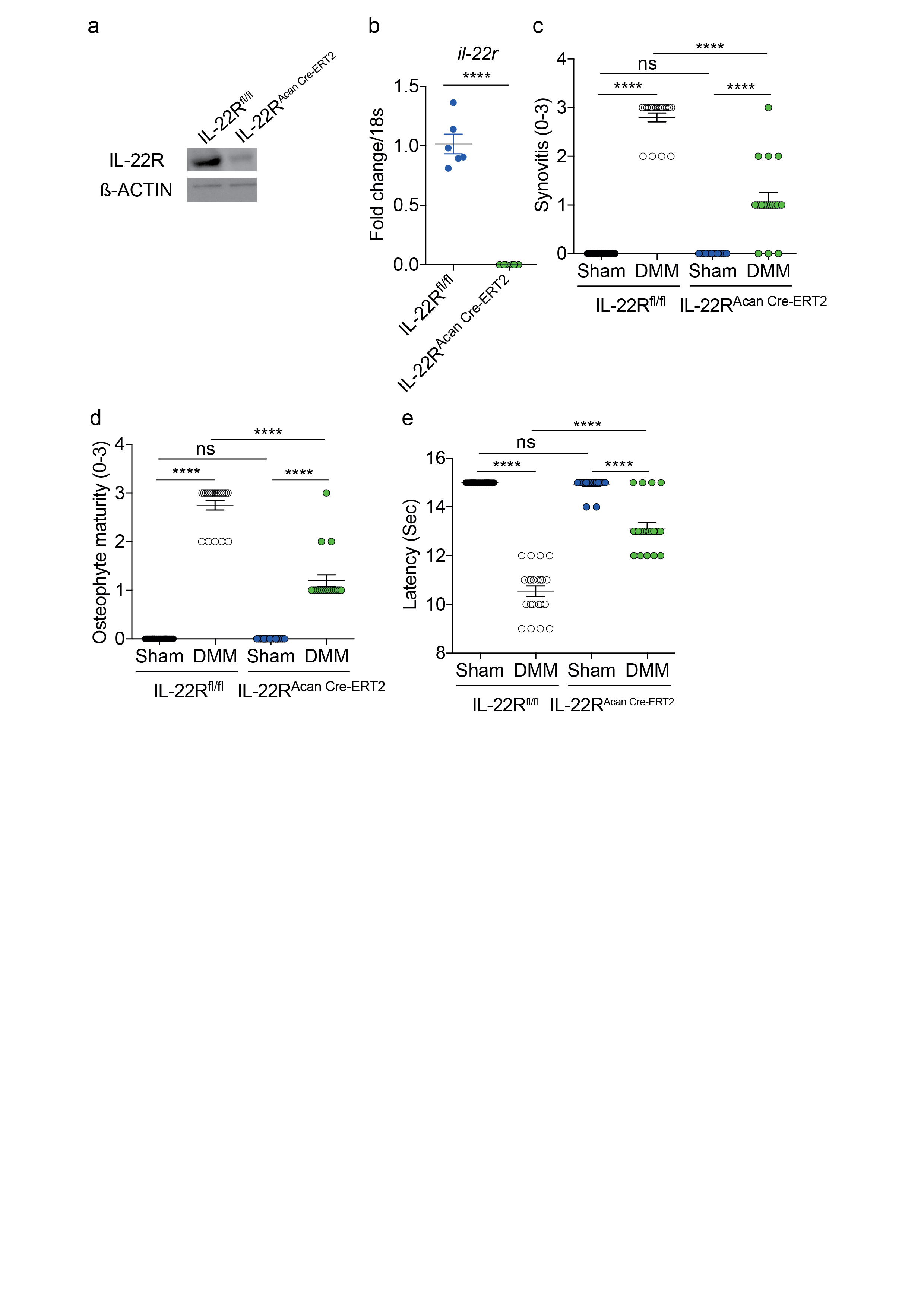


**Supplementary figure 1: Validation of IL-22^ACAN CRE-ERT2^ KO mice and disease outcomes 12 weeks post DMM surgery. (a)** Protein expression of IL-22R from cartilaginous hips avulsed from young (7 weeks old) IL-22R^Acan Cre-ERT2^ and IL-22R^fl/fl^ control mice. **(b)** mRNA expression in microdissected articular cartilage from knees joints obtained from IL-22R^Acan Cre-ERT2^ and IL-22R^fl/fl^ control mice (10 weeks old mice; *n*=6). **(c)** synovitis scoring and **(d)** hot plate pain assay in sham- or DMM-operated IL-22R^fl/fl^ control mice and IL-22R^Acan Cre-ERT2^ mice (12 weeks post-surgery end timepoint) (*n*=20). All RT-qPCR gene expressions were normalized to the endogenous level of 18s in respective groups. Data are expressed as mean ± S.E.M with two-tailed *t*-test or analysed with two-way analysis of variance followed by the Tukey-Kramer test. *n* indicates the number of human specimens or mice per group. NS= non-significant. p< 0.0001 represented in all figures as **** respectively.

**
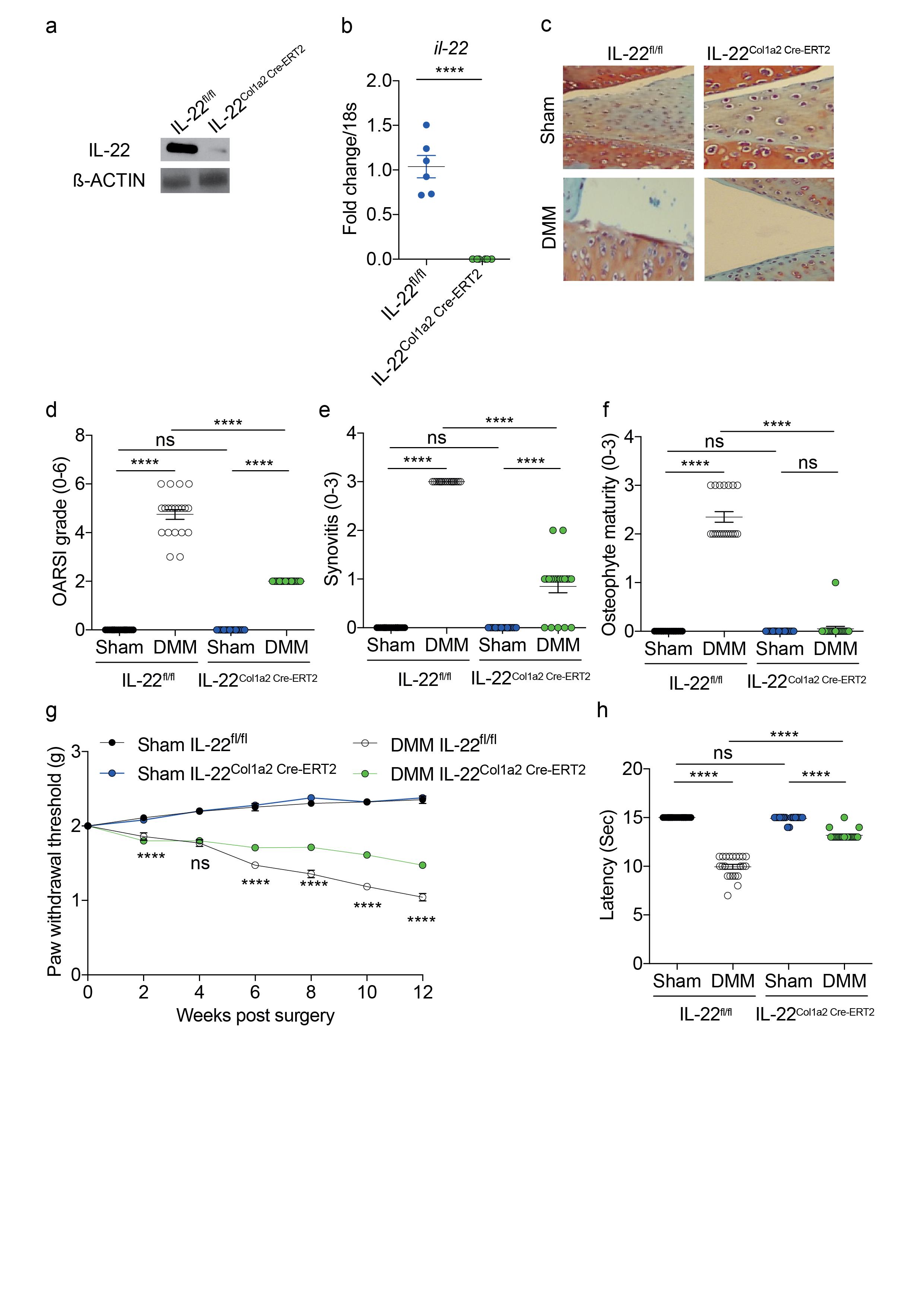
**

**Supplementary figure 2: Validation of** IL-22^Col1a2 Cre-ERT2^ **KO mice and disease outcomes 12 weeks post DMM surgery. (a)** Protein expression of IL-22 in FLS obtained from (10 weeks old) IL-22^Col1a2 Cre-ERT2^ and IL-22^fl/fl^ control mice. **(b)** IL-22 mRNA expression in FLS obtained from IL-22^Col1a2 Cre-ERT2^ mice and IL-22^fl/fl^ control mice (10 weeks old mice; *n*=6). **(c and d)** OARSI scoring of cartilage **(e)** synovitis scoring **(f)** osteophyte maturity scoring **(g)** von Frey pain assessment **(h)** Hot plate pain assay of sham- (*n*=20) or DMM- (*n*=20) operated IL-22^fl/fl^ control mice and IL-22^Col1a2 Cre-ERT2^ mice (12 weeks post-surgery end timepoint). All RT-qPCR gene expressions were normalized to the endogenous level of 18s in respective groups. Data are expressed as mean ± S.E.M with two-tailed *t*-test or analysed with two-way analysis of variance followed by the Tukey-Kramer test or repeated measures 2-way ANOVA with Bonferroni’s post hoc tests. *n* indicates the number of human specimens or mice per group. NS= non-significant. p< 0.0001 represented in all figures as **** respectively.

**
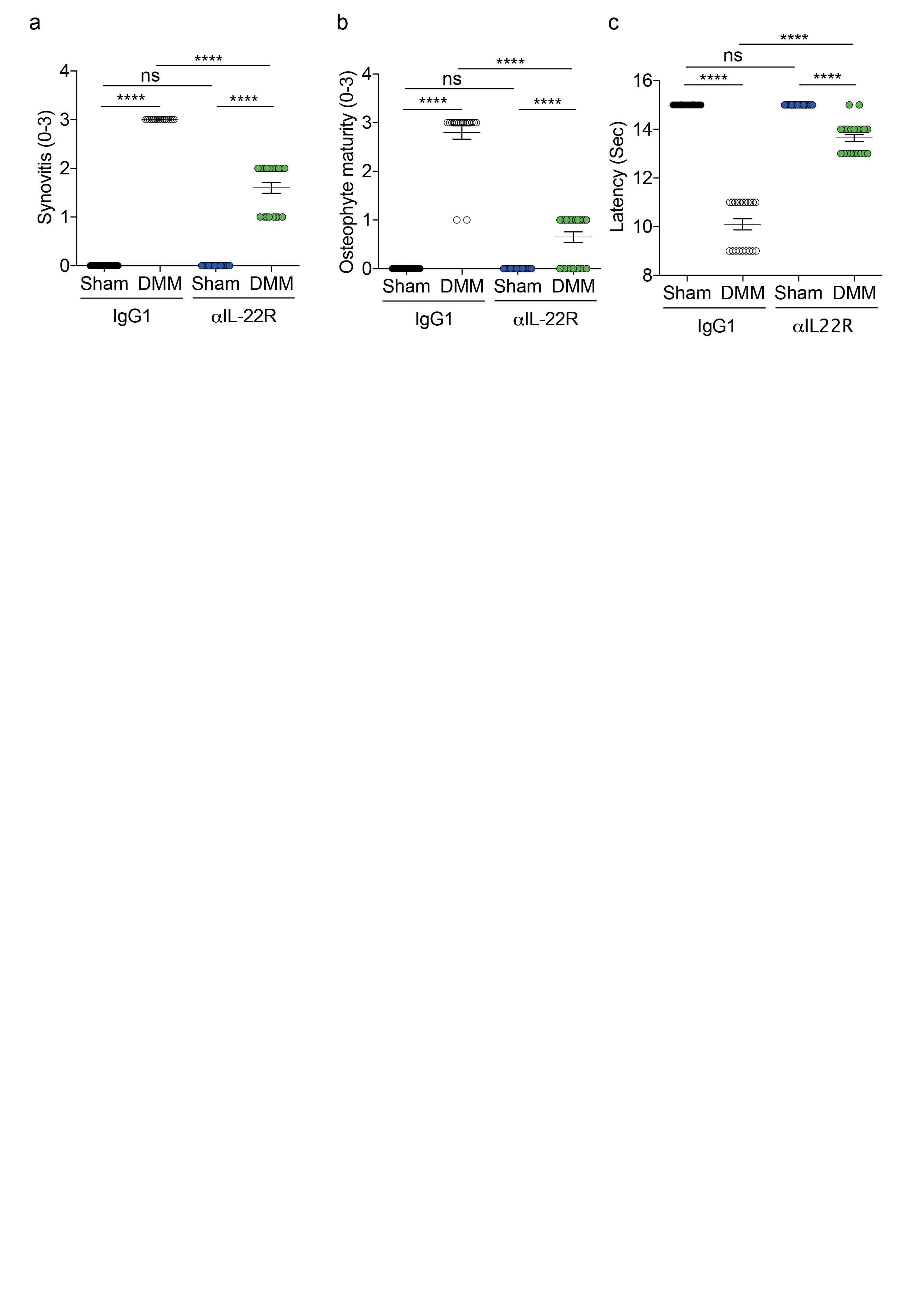
**

**Supplementary figure 3: Determination of disease outcomes in mice treated with αIL-22R 12 weeks post DMM surgery. (a)** synovitis scoring and **(b)** osteophyte maturity scoring **(c)** hot plate pain assay from sham- (*n*=20) or DMM- (*n*=20) operated wildtype mice (12 weeks post-surgery end timepoint) treated intra-articularly with either IgG1 either IgG1 (vehicle control; 50 µg per mouse; 3 times per week for 12 weeks post-surgery) or αIL-22R (50 µg per mouse; 3 times per week for 12 weeks post-surgery). Data are expressed as mean ± S.E.M with two-way analysis of variance followed by the Tukey-Kramer test. *n* indicates the number of human specimens or mice per group. NS= non-significant. p< 0.0001 represented in all figures as **** respectively.

**
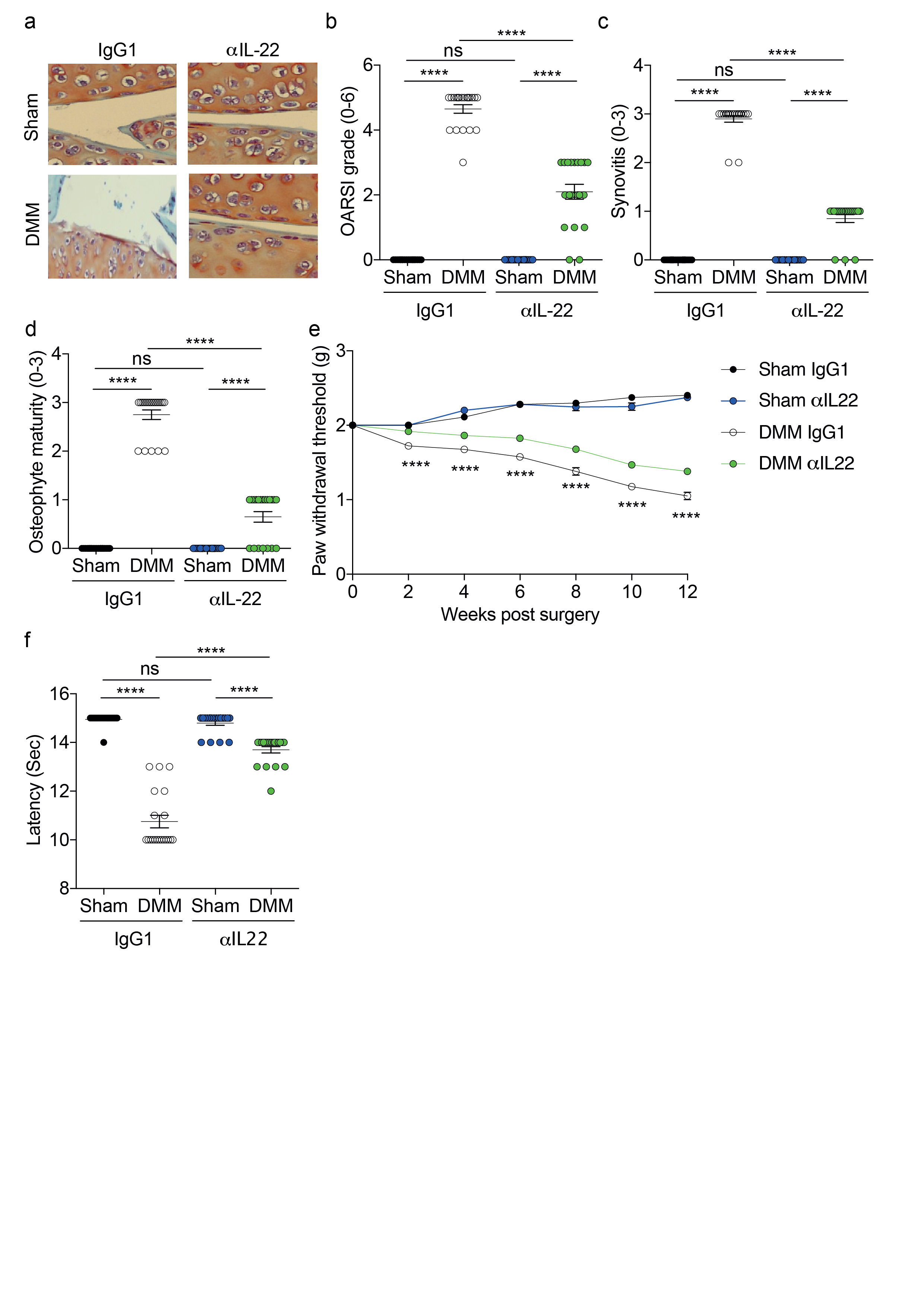
**

**Supplementary figure 4: Determination of disease outcomes in mice treated with αIL-22 12 weeks post DMM surgery. (a and b)** OARSI scoring of cartilage **(c)** synovitis scoring and **(d)** osteophyte maturity scoring **(e)** von Frey pain assessment **(f)** hot plate pain assay from sham- or DMM-operated WT mice treated i.a. with either IgG1 (control; 50 µg per mouse; 3 times per week for 12 weeks post-surgery) or αIL-22 (50 µg per mouse; 3 times per week for 12 weeks post-surgery). (*n*=20). Data are expressed as mean ± S.E.M with two-way analysis of variance followed by the Tukey-Kramer test or repeated measures 2-way ANOVA with Bonferroni’s post hoc tests. *n* indicates the number of human specimens or mice per group. NS= non-significant. p< 0.0001 represented in all figures as **** respectively.
